# Supplementary material for: Pangenome-wide association study reveals the selective absence of CRISPR genes (Rv2816c-19c) in drug-resistant Mycobacterium tuberculosis
Source: Microbiol Spectr. 2024 Jun 25;12(8):e00527-24. doi: 10.1128/spectrum.00527-24 (PMC11302280; doi:10.1128/spectrum.00527-24)
Supplement: Supplemental material — Fig. S1 to S3; Tables S1 to S3. [file spectrum.00527-24-s0001.pdf]

Supplementary Information.

Pangenome-wide association study reveals the selective absence of CRISPR genes (Rv2816c-19c) in drug-resistant *Mycobacterium tuberculosis.*

Nikhil Bhalla, Ranjan Kumar Nanda

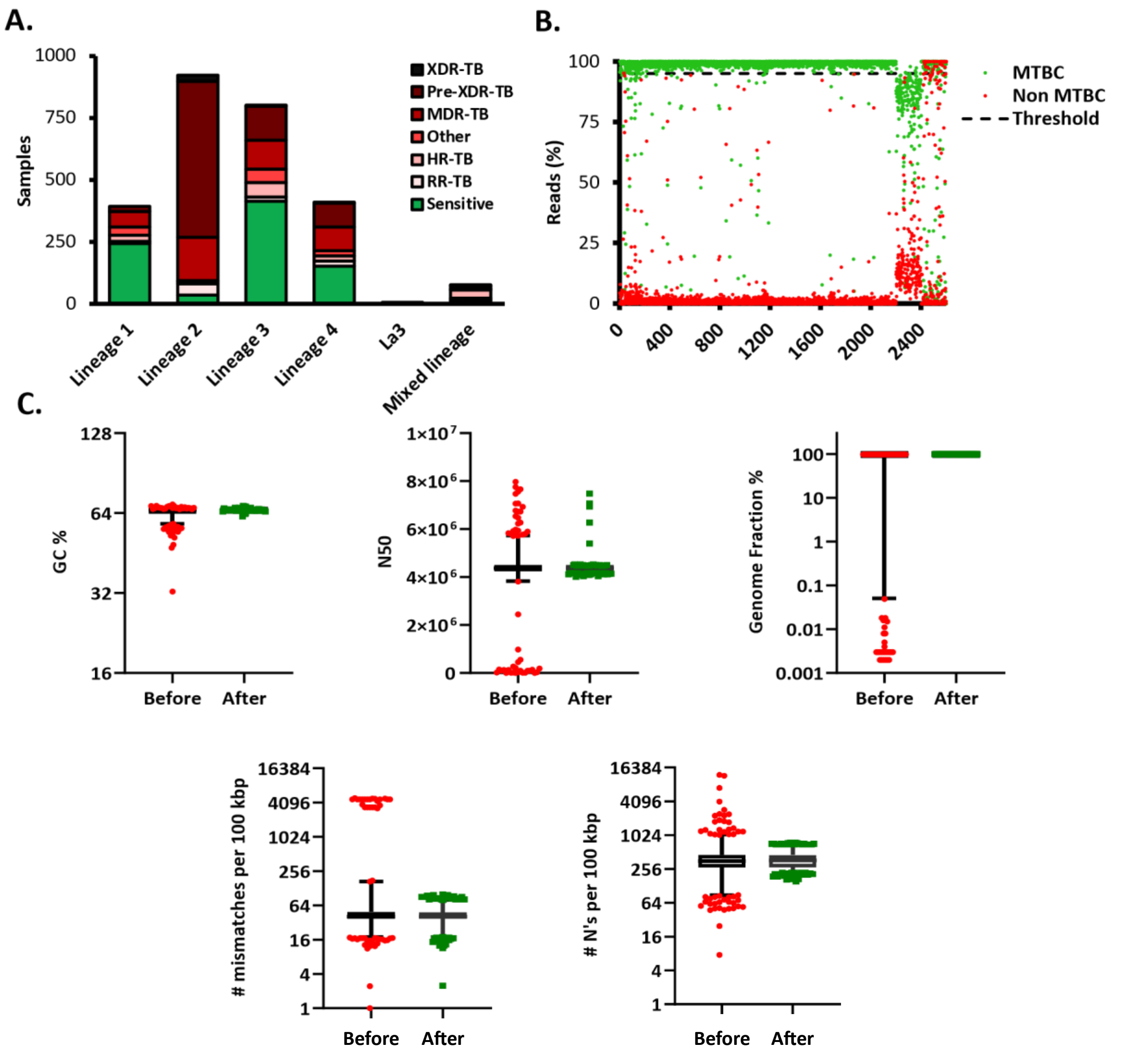

Supplementary Figure S1: QC of WGS data and filtering. A. Percentage and Number of drug-resistant and drug-susceptible Mtb isolates. **B**. Metagenome determination in WGS data. The X-axis shows the percentage of reads aligning to MTBC (Green) and Unclassified + Non-MTBC (Red). Samples having 95% reads aligning to MTBC species were retained (Black line threshold). **C**. Quality metrics of de novo scaffolds before and after filtering (GC % > 62; N50 > 3999999; Genome Fraction % > 95; Mismatches per 100Kbp < 100).

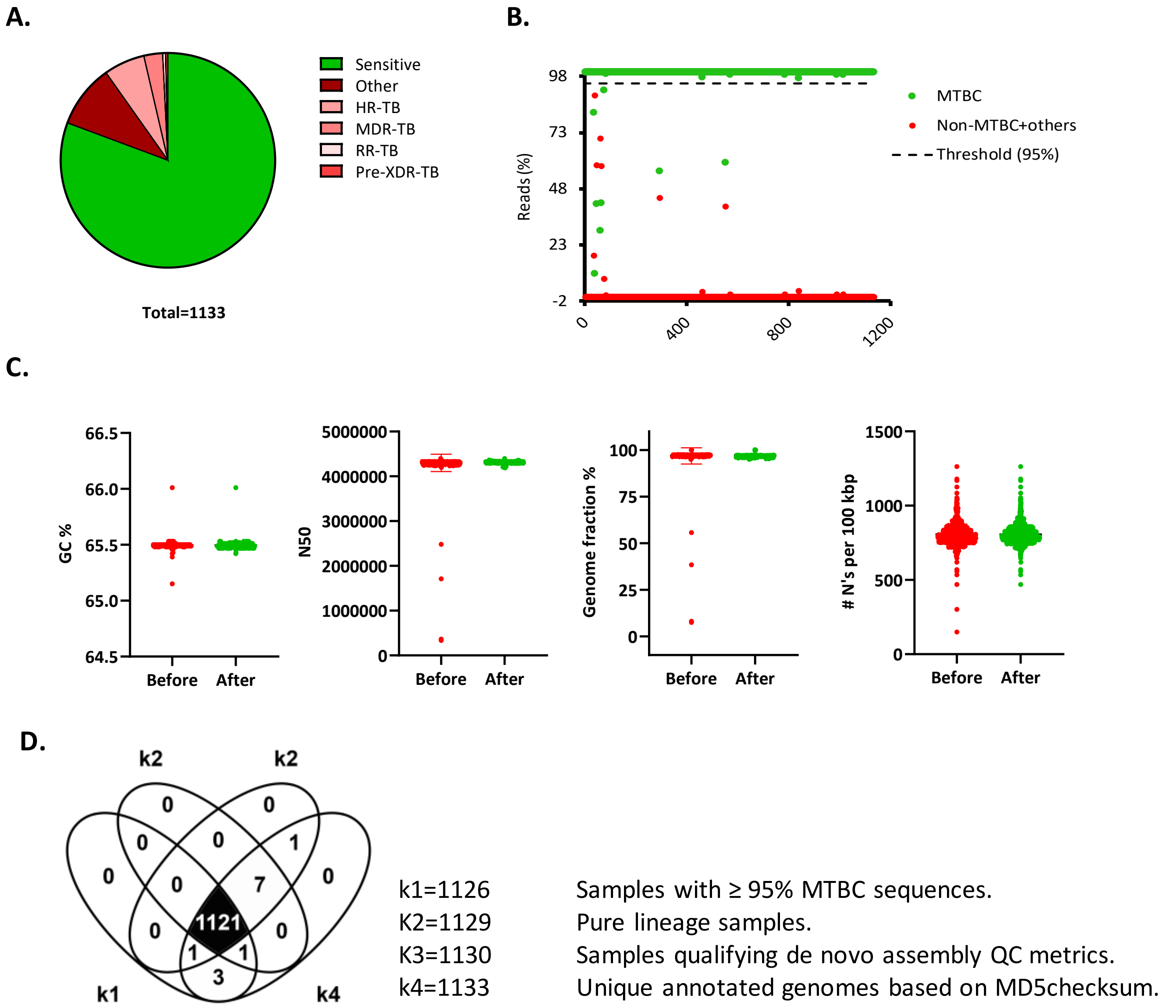

Supplementary Figure S2: Data processing of Mtb WGS data from a non-endemic region (Netherlands). A. Samples constituting drug-resistant and sensitive groups. **C.** *De novo* quality of assemblies before and after filtering. **B**. Metagenome detection in WGS datasets. The graph shows percentage of MTBC, Non-MTBC plus unclassified sequences. **D.** Venn analysis to filter samples having negligible contamination, were unmixed/pure lineage samples, qualified *de novo* quality filtering and are unique.

A.

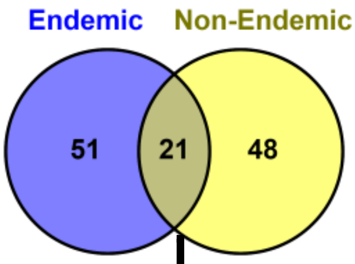

B. 21 genes having selective presence in drug resistance in endemic and non-endemic regions.

Locus tag	Product	$Log_{10}OR$	
		Endemic	Non-endemic
Rv0071	maturase	-1.41	-0.47
Rv0072	glutamine ABC transporter permease	-1.37	0.48
Rv0073	glutamine ABC transporter ATP-binding protein	-1.40	-0.50
Rv0394c	hypothetical protein	0.62	-1.06
Rv1255c	HTH-type transcriptional regulator	0.84	-1.37
Rv1967	Mce family protein Mce3B	-0.80	-0.89
Rv2645	hypothetical protein	0.62	-0.52
Rv2646	probable integrase	0.61	-0.52
Rv2650c	prophage protein	0.60	-0.54
Rv2652c	prophage protein	0.58	-0.43
Rv2653c	toxin	0.62	-0.52
Rv2654c	antitoxin	0.57	-0.50
Rv2655c	prophage protein	0.61	-0.52
Rv2656c	prophage protein	0.62	-0.53
Rv2657c	prophage protein	0.62	-0.52
Rv2658c	prophage protein	0.62	-0.52
Rv2659c	prophage integrase	0.61	-0.52
Rv2816c	CRISPR-associated endoribonuclease Cas2	-1.30	-0.42
Rv2817c	CRISPR-associated endonuclease Cas1	-1.04	-0.40
Rv2818c	CRISPR-associated protein Csm6	-1.29	0.75
Rv3919c	16S rRNA (guanine(527)-N(7))-methyltransferase RsmG	2.22	2.11

C. 6 genes having similar presence-absence in drug resistance Mtb isolates in endemic and non-endemic regions.

Locus tag	Product	$Log_{10}OR$	
		Endemic	Non-endemic
Rv0071	maturase	-1.41	-0.47
Rv0073	glutamine ABC transporter ATP-binding protein	-1.40	-0.50
Rv1967	Mce family protein Mce3B	-0.80	-0.89
Rv2816c	CRISPR-associated endoribonuclease Cas2	-1.30	-0.42
Rv2817c	CRISPR-associated endonuclease Cas1	-1.04	-0.40
Rv3919c	16S rRNA (guanine(527)-N(7))-methyltransferase RsmG	2.22	2.11

Supplementary Figure S3: Comparing genes with selective presence/absence in drug-resistant Mtb isolates from TB endemic and non-endemic regions. A. Venn analysis of genes with selective presence/absence in drug-resistant Mtb isolates from TB-endemic and non-endemic regions. **B.** Genes with differential presence in drug-resistant Mtb in both TB-endemic and non-endemic regions. **C.** Genes with similar presence-absence patterns in TB-endemic and non-endemic regions.

Supplementary Table S1: SRA run IDs of the high-quality WGS dataset (n=2184) from TB-endemic regions (India, Pakistan, Zambia and China). The annotated assemblies of these WGS datasets were used for Pan-GWAS analysis. SRA run ID marked in red are drug-resistant and those in black are sensitive.

ERR3077930	ERR3335747	ERR4796285	ERR4796396	ERR4796509	ERR4796601	ERR4796710
ERR3077932	ERR3335748	ERR4796286	ERR4796397	ERR4796510	ERR4796602	ERR4796711
ERR3077937	ERR3335749	ERR4796287	ERR4796398	ERR4796511	ERR4796605	ERR4796712
ERR3077938	ERR3335750	ERR4796289	ERR4796399	ERR4796512	ERR4796607	ERR4796714
ERR3077939	ERR3335751	ERR4796291	ERR4796404	ERR4796513	ERR4796608	ERR4796715
ERR3077940	ERR3335752	ERR4796294	ERR4796405	ERR4796514	ERR4796609	ERR4796718
ERR3077941	ERR3335754	ERR4796297	ERR4796408	ERR4796516	ERR4796612	ERR4796722
ERR3077942	ERR3335757	ERR4796303	ERR4796409	ERR4796517	ERR4796613	ERR4796723
ERR3077943	ERR3335761	ERR4796304	ERR4796410	ERR4796518	ERR4796615	ERR4796724
ERR3077944	ERR3335763	ERR4796306	ERR4796412	ERR4796519	ERR4796618	ERR4796725
ERR3077945	ERR3335764	ERR4796307	ERR4796413	ERR4796521	ERR4796621	ERR4796726
ERR3077950	ERR3335766	ERR4796309	ERR4796414	ERR4796522	ERR4796622	ERR4796727
ERR3077954	ERR3335767	ERR4796311	ERR4796415	ERR4796523	ERR4796623	ERR4796729
ERR3077955	ERR3335769	ERR4796314	ERR4796416	ERR4796524	ERR4796624	ERR4796730
ERR3077956	ERR3335771	ERR4796315	ERR4796418	ERR4796525	ERR4796627	ERR4796732
ERR3077958	ERR3335772	ERR4796316	ERR4796420	ERR4796526	ERR4796628	ERR4796733
ERR3077961	ERR3335773	ERR4796319	ERR4796421	ERR4796527	ERR4796629	ERR4796734
ERR3077962	ERR3335774	ERR4796324	ERR4796423	ERR4796528	ERR4796631	ERR4796735
ERR3077964	ERR3335775	ERR4796326	ERR4796424	ERR4796529	ERR4796633	ERR4796737
ERR3077965	ERR3335776	ERR4796327	ERR4796425	ERR4796532	ERR4796634	ERR4796740
ERR3077967	ERR3335777	ERR4796328	ERR4796427	ERR4796533	ERR4796635	ERR4796742
ERR3077970	ERR3335778	ERR4796329	ERR4796430	ERR4796534	ERR4796636	ERR4796743
ERR3077971	ERR3335779	ERR4796331	ERR4796432	ERR4796536	ERR4796641	ERR4796744
ERR3077975	ERR3335780	ERR4796333	ERR4796434	ERR4796538	ERR4796645	ERR4796745
ERR3077980	ERR3335781	ERR4796335	ERR4796435	ERR4796541	ERR4796646	ERR4796746
ERR3077985	ERR3335782	ERR4796337	ERR4796436	ERR4796542	ERR4796647	ERR4796749
ERR3077987	ERR3335784	ERR4796339	ERR4796438	ERR4796543	ERR4796648	ERR4796750
ERR3077990	ERR3335785	ERR4796341	ERR4796439	ERR4796544	ERR4796649	ERR4796751
ERR3077992	ERR3335786	ERR4796342	ERR4796440	ERR4796545	ERR4796652	ERR4796754
ERR3077994	ERR3335787	ERR4796343	ERR4796441	ERR4796547	ERR4796654	ERR4796757
ERR3077996	ERR3335788	ERR4796344	ERR4796442	ERR4796548	ERR4796655	ERR4796758
ERR3077997	ERR3335789	ERR4796346	ERR4796443	ERR4796549	ERR4796657	ERR4796760
ERR3077998	ERR3335790	ERR4796349	ERR4796444	ERR4796553	ERR4796659	ERR4796761
ERR3077999	ERR3335791	ERR4796350	ERR4796447	ERR4796555	ERR4796660	ERR4796763
ERR3078003	ERR3335792	ERR4796352	ERR4796448	ERR4796557	ERR4796661	ERR4796764
ERR3078004	ERR3335793	ERR4796353	ERR4796449	ERR4796558	ERR4796662	ERR4796765
ERR3078005	ERR3335794	ERR4796355	ERR4796450	ERR4796559	ERR4796663	ERR4796767
ERR3078006	ERR3335797	ERR4796357	ERR4796451	ERR4796560	ERR4796665	ERR4796768
ERR3078007	ERR3335799	ERR4796359	ERR4796452	ERR4796561	ERR4796667	ERR4796771
ERR3078013	ERR3335800	ERR4796360	ERR4796454	ERR4796562	ERR4796672	ERR4796773
ERR3078014	ERR3335801	ERR4796361	ERR4796456	ERR4796565	ERR4796673	ERR4796774
ERR3078016	ERR4796256	ERR4796365	ERR4796458	ERR4796566	ERR4796676	ERR4796775
ERR3078017	ERR4796259	ERR4796368	ERR4796462	ERR4796568	ERR4796677	ERR4796776
ERR3335723	ERR4796260	ERR4796369	ERR4796463	ERR4796570	ERR4796679	ERR4796777
ERR3335724	ERR4796261	ERR4796371	ERR4796466	ERR4796571	ERR4796681	ERR4796779
ERR3335726	ERR4796262	ERR4796372	ERR4796467	ERR4796572	ERR4796682	ERR4796780
ERR3335728	ERR4796263	ERR4796374	ERR4796468	ERR4796573	ERR4796683	ERR4796781
ERR3335730	ERR4796264	ERR4796375	ERR4796469	ERR4796575	ERR4796689	ERR4796782
ERR3335731	ERR4796265	ERR4796377	ERR4796473	ERR4796576	ERR4796690	ERR4796785
ERR3335732	ERR4796266	ERR4796378	ERR4796478	ERR4796578	ERR4796692	ERR4796786
ERR3335733	ERR4796267	ERR4796379	ERR4796479	ERR4796579	ERR4796694	ERR4796787
ERR3335734	ERR4796269	ERR4796380	ERR4796481	ERR4796581	ERR4796695	ERR4796788
ERR3335736	ERR4796270	ERR4796382	ERR4796483	ERR4796585	ERR4796697	ERR4796789
ERR3335737	ERR4796271	ERR4796383	ERR4796484	ERR4796586	ERR4796698	ERR4796790
ERR3335738	ERR4796272	ERR4796385	ERR4796486	ERR4796588	ERR4796699	ERR4796791
ERR3335739	ERR4796273	ERR4796387	ERR4796488	ERR4796589	ERR4796700	ERR4796792
ERR3335740	ERR4796274	ERR4796388	ERR4796490	ERR4796590	ERR4796701	ERR4796793
ERR3335741	ERR4796275	ERR4796389	ERR4796491	ERR4796594	ERR4796702	ERR4796794
ERR3335742	ERR4796276	ERR4796390	ERR4796496	ERR4796595	ERR4796703	ERR4796795
ERR3335743	ERR4796277	ERR4796391	ERR4796497	ERR4796596	ERR4796704	ERR4796797
ERR3335744	ERR4796278	ERR4796392	ERR4796502	ERR4796597	ERR4796706	ERR4796798
ERR3335745	ERR4796281	ERR4796394	ERR4796503	ERR4796599	ERR4796707	ERR4796802
ERR3335746	ERR4796282	ERR4796395	ERR4796506	ERR4796600	ERR4796709	ERR4796803

ERR4796804	ERR4796918	ERR4797027	ERR4797181	ERR4797333	ERR4797478	ERR4797594
ERR4796805	ERR4796921	ERR4797028	ERR4797183	ERR4797334	ERR4797479	ERR4797596
ERR4796807	ERR4796922	ERR4797035	ERR4797185	ERR4797337	ERR4797481	ERR4797597
ERR4796808	ERR4796923	ERR4797040	ERR4797186	ERR4797338	ERR4797483	ERR4797600
ERR4796812	ERR4796924	ERR4797043	ERR4797191	ERR4797340	ERR4797485	ERR4797601
ERR4796813	ERR4796925	ERR4797045	ERR4797192	ERR4797341	ERR4797486	ERR4797604
ERR4796816	ERR4796926	ERR4797046	ERR4797194	ERR4797343	ERR4797489	ERR4797606
ERR4796817	ERR4796927	ERR4797048	ERR4797195	ERR4797346	ERR4797490	ERR4797607
ERR4796818	ERR4796928	ERR4797051	ERR4797196	ERR4797349	ERR4797491	ERR4797608
ERR4796820	ERR4796930	ERR4797052	ERR4797199	ERR4797351	ERR4797492	ERR4797611
ERR4796821	ERR4796931	ERR4797053	ERR4797200	ERR4797353	ERR4797497	ERR4797612
ERR4796822	ERR4796933	ERR4797056	ERR4797203	ERR4797355	ERR4797498	ERR4797613
ERR4796823	ERR4796936	ERR4797059	ERR4797204	ERR4797356	ERR4797499	ERR4797617
ERR4796824	ERR4796937	ERR4797060	ERR4797208	ERR4797358	ERR4797501	ERR4797618
ERR4796825	ERR4796938	ERR4797066	ERR4797209	ERR4797359	ERR4797502	ERR4797620
ERR4796826	ERR4796939	ERR4797068	ERR4797210	ERR4797360	ERR4797503	ERR4797621
ERR4796827	ERR4796940	ERR4797070	ERR4797213	ERR4797363	ERR4797504	ERR4797622
ERR4796828	ERR4796942	ERR4797072	ERR4797214	ERR4797366	ERR4797506	ERR4797626
ERR4796831	ERR4796944	ERR4797075	ERR4797215	ERR4797367	ERR4797507	ERR4797629
ERR4796832	ERR4796945	ERR4797080	ERR4797217	ERR4797370	ERR4797508	ERR4797631
ERR4796833	ERR4796946	ERR4797081	ERR4797218	ERR4797373	ERR4797510	ERR4797632
ERR4796836	ERR4796947	ERR4797082	ERR4797219	ERR4797377	ERR4797511	ERR4797633
ERR4796837	ERR4796948	ERR4797083	ERR4797221	ERR4797378	ERR4797512	ERR4797634
ERR4796838	ERR4796949	ERR4797088	ERR4797223	ERR4797380	ERR4797514	ERR4797635
ERR4796839	ERR4796950	ERR4797090	ERR4797227	ERR4797382	ERR4797516	ERR4797636
ERR4796840	ERR4796952	ERR4797094	ERR4797228	ERR4797384	ERR4797518	ERR4797637
ERR4796843	ERR4796953	ERR4797097	ERR4797230	ERR4797387	ERR4797521	ERR4797638
ERR4796845	ERR4796954	ERR4797099	ERR4797232	ERR4797389	ERR4797522	ERR4797640
ERR4796847	ERR4796955	ERR4797102	ERR4797234	ERR4797390	ERR4797523	ERR4797641
ERR4796849	ERR4796956	ERR4797105	ERR4797235	ERR4797391	ERR4797524	ERR4797642
ERR4796850	ERR4796958	ERR4797106	ERR4797246	ERR4797392	ERR4797525	ERR4797643
ERR4796851	ERR4796959	ERR4797107	ERR4797247	ERR4797393	ERR4797526	ERR4797644
ERR4796855	ERR4796960	ERR4797108	ERR4797263	ERR4797394	ERR4797528	ERR4797645
ERR4796857	ERR4796961	ERR4797110	ERR4797265	ERR4797395	ERR4797529	ERR4797648
ERR4796859	ERR4796962	ERR4797111	ERR4797266	ERR4797399	ERR4797531	ERR4797651
ERR4796861	ERR4796963	ERR4797113	ERR4797268	ERR4797400	ERR4797535	ERR4797652
ERR4796862	ERR4796964	ERR4797114	ERR4797269	ERR4797401	ERR4797537	ERR4797653
ERR4796863	ERR4796965	ERR4797115	ERR4797272	ERR4797402	ERR4797539	ERR4797657
ERR4796864	ERR4796967	ERR4797117	ERR4797274	ERR4797405	ERR4797542	ERR4797658
ERR4796868	ERR4796968	ERR4797119	ERR4797275	ERR4797406	ERR4797544	ERR4797660
ERR4796869	ERR4796972	ERR4797121	ERR4797277	ERR4797407	ERR4797545	ERR4797661
ERR4796871	ERR4796973	ERR4797124	ERR4797278	ERR4797408	ERR4797547	ERR4797662
ERR4796872	ERR4796974	ERR4797126	ERR4797279	ERR4797409	ERR4797549	ERR4797663
ERR4796875	ERR4796975	ERR4797128	ERR4797287	ERR4797412	ERR4797550	ERR4797665
ERR4796876	ERR4796976	ERR4797130	ERR4797288	ERR4797413	ERR4797551	ERR4797666
ERR4796877	ERR4796977	ERR4797131	ERR4797289	ERR4797414	ERR4797552	ERR4797668
ERR4796878	ERR4796978	ERR4797132	ERR4797290	ERR4797415	ERR4797553	ERR4797669
ERR4796879	ERR4796979	ERR4797133	ERR4797291	ERR4797419	ERR4797555	ERR4797672
ERR4796881	ERR4796982	ERR4797137	ERR4797292	ERR4797421	ERR4797556	ERR4797673
ERR4796882	ERR4796983	ERR4797143	ERR4797295	ERR4797423	ERR4797557	ERR4797674
ERR4796884	ERR4796984	ERR4797144	ERR4797296	ERR4797426	ERR4797558	ERR4797676
ERR4796885	ERR4796986	ERR4797145	ERR4797297	ERR4797428	ERR4797560	ERR4797677
ERR4796890	ERR4796987	ERR4797147	ERR4797301	ERR4797432	ERR4797561	ERR4797678
ERR4796891	ERR4796989	ERR4797148	ERR4797302	ERR4797434	ERR4797562	ERR4797679
ERR4796892	ERR4796990	ERR4797150	ERR4797303	ERR4797436	ERR4797563	ERR4797680
ERR4796893	ERR4796992	ERR4797151	ERR4797304	ERR4797438	ERR4797564	ERR4797682
ERR4796894	ERR4796995	ERR4797152	ERR4797305	ERR4797439	ERR4797565	ERR4797683
ERR4796896	ERR4796997	ERR4797157	ERR4797307	ERR4797440	ERR4797566	ERR4797687
ERR4796897	ERR4796998	ERR4797158	ERR4797310	ERR4797441	ERR4797567	ERR4797688
ERR4796898	ERR4796999	ERR4797160	ERR4797314	ERR4797445	ERR4797570	ERR4797689
ERR4796903	ERR4797000	ERR4797162	ERR4797315	ERR4797451	ERR4797573	ERR4797690
ERR4796905	ERR4797001	ERR4797163	ERR4797317	ERR4797453	ERR4797577	ERR4797691
ERR4796906	ERR4797007	ERR4797166	ERR4797319	ERR4797454	ERR4797578	ERR4797692
ERR4796907	ERR4797008	ERR4797167	ERR4797321	ERR4797457	ERR4797579	ERR4797693
ERR4796908	ERR4797014	ERR4797171	ERR4797322	ERR4797458	ERR4797580	ERR4797695
ERR4796910	ERR4797015	ERR4797172	ERR4797323	ERR4797459	ERR4797581	ERR4797696
ERR4796912	ERR4797016	ERR4797173	ERR4797324	ERR4797466	ERR4797582	ERR4797697
ERR4796913	ERR4797017	ERR4797174	ERR4797325	ERR4797467	ERR4797586	ERR4797702
ERR4796914	ERR4797018	ERR4797175	ERR4797326	ERR4797468	ERR4797587	ERR4797703
ERR4796916	ERR4797020	ERR4797178	ERR4797329	ERR4797469	ERR4797591	ERR4797704
ERR4796917	ERR4797025	ERR4797180	ERR4797331	ERR4797471	ERR4797592	ERR4797705

ERR4797709	ERR4797823	ERR4797944	ERR4798068	ERR4798196	ERR4798443	SRR5341398
ERR4797710	ERR4797824	ERR4797945	ERR4798069	ERR4798198	ERR4798838	SRR5341399
ERR4797711	ERR4797826	ERR4797946	ERR4798070	ERR4798200	ERR4798839	SRR5341402
ERR4797712	ERR4797827	ERR4797951	ERR4798073	ERR4798201	ERR4798840	SRR5341403
ERR4797713	ERR4797828	ERR4797952	ERR4798075	ERR4798202	ERR4799552	SRR5341409
ERR4797714	ERR4797829	ERR4797953	ERR4798079	ERR4798204	ERR4799553	SRR5341410
ERR4797715	ERR4797830	ERR4797955	ERR4798080	ERR4798205	SRR21576540	SRR5341416
ERR4797716	ERR4797831	ERR4797957	ERR4798081	ERR4798207	SRR21576610	SRR5341433
ERR4797719	ERR4797834	ERR4797961	ERR4798082	ERR4798208	SRR21576619	SRR5341434
ERR4797720	ERR4797836	ERR4797962	ERR4798085	ERR4798209	SRR21576645	SRR5341436
ERR4797722	ERR4797843	ERR4797963	ERR4798087	ERR4798210	SRR21576648	SRR5341441
ERR4797725	ERR4797844	ERR4797965	ERR4798090	ERR4798212	SRR21576689	SRR5341444
ERR4797726	ERR4797846	ERR4797967	ERR4798091	ERR4798216	SRR21576694	SRR5341445
ERR4797727	ERR4797847	ERR4797968	ERR4798100	ERR4798219	SRR21576697	SRR5341446
ERR4797732	ERR4797849	ERR4797971	ERR4798102	ERR4798221	SRR21576730	SRR5341448
ERR4797735	ERR4797850	ERR4797973	ERR4798107	ERR4798224	SRR5341272	SRR5341451
ERR4797738	ERR4797852	ERR4797975	ERR4798110	ERR4798225	SRR5341273	SRR5341457
ERR4797739	ERR4797854	ERR4797979	ERR4798111	ERR4798226	SRR5341274	SRR5341458
ERR4797740	ERR4797856	ERR4797980	ERR4798112	ERR4798229	SRR5341275	SRR5341464
ERR4797742	ERR4797857	ERR4797981	ERR4798113	ERR4798230	SRR5341276	SRR5341466
ERR4797747	ERR4797858	ERR4797982	ERR4798114	ERR4798231	SRR5341277	SRR5341468
ERR4797748	ERR4797859	ERR4797983	ERR4798115	ERR4798232	SRR5341279	SRR5341471
ERR4797750	ERR4797860	ERR4797986	ERR4798116	ERR4798234	SRR5341280	ERR3077934
ERR4797751	ERR4797861	ERR4797987	ERR4798119	ERR4798235	SRR5341281	ERR3077935
ERR4797753	ERR4797864	ERR4797988	ERR4798120	ERR4798238	SRR5341283	ERR3077936
ERR4797754	ERR4797865	ERR4797992	ERR4798122	ERR4798239	SRR5341284	ERR3077946
ERR4797755	ERR4797868	ERR4797993	ERR4798124	ERR4798240	SRR5341285	ERR3077949
ERR4797756	ERR4797869	ERR4797994	ERR4798126	ERR4798242	SRR5341286	ERR3077951
ERR4797757	ERR4797870	ERR4797995	ERR4798127	ERR4798243	SRR5341292	ERR3077952
ERR4797758	ERR4797871	ERR4797997	ERR4798128	ERR4798246	SRR5341294	ERR3077953
ERR4797759	ERR4797872	ERR4797998	ERR4798129	ERR4798247	SRR5341304	ERR3077959
ERR4797760	ERR4797875	ERR4797999	ERR4798130	ERR4798248	SRR5341305	ERR3077973
ERR4797762	ERR4797876	ERR4798003	ERR4798131	ERR4798249	SRR5341307	ERR3077974
ERR4797763	ERR4797878	ERR4798004	ERR4798133	ERR4798250	SRR5341309	ERR3077976
ERR4797764	ERR4797880	ERR4798005	ERR4798134	ERR4798251	SRR5341311	ERR3077977
ERR4797765	ERR4797883	ERR4798006	ERR4798135	ERR4798252	SRR5341314	ERR3077981
ERR4797770	ERR4797884	ERR4798007	ERR4798136	ERR4798254	SRR5341318	ERR3077982
ERR4797772	ERR4797885	ERR4798008	ERR4798138	ERR4798255	SRR5341320	ERR3077983
ERR4797776	ERR4797893	ERR4798009	ERR4798140	ERR4798256	SRR5341321	ERR3077984
ERR4797777	ERR4797894	ERR4798010	ERR4798141	ERR4798257	SRR5341322	ERR3077986
ERR4797778	ERR4797896	ERR4798012	ERR4798142	ERR4798259	SRR5341325	ERR3077988
ERR4797779	ERR4797897	ERR4798015	ERR4798143	ERR4798260	SRR5341326	ERR3077993
ERR4797780	ERR4797898	ERR4798016	ERR4798144	ERR4798261	SRR5341327	ERR3078001
ERR4797783	ERR4797902	ERR4798017	ERR4798146	ERR4798264	SRR5341328	ERR3078002
ERR4797786	ERR4797903	ERR4798018	ERR4798149	ERR4798265	SRR5341329	ERR3078009
ERR4797788	ERR4797904	ERR4798022	ERR4798150	ERR4798268	SRR5341338	ERR3078011
ERR4797789	ERR4797906	ERR4798023	ERR4798152	ERR4798269	SRR5341339	ERR3078012
ERR4797791	ERR4797908	ERR4798025	ERR4798153	ERR4798270	SRR5341347	ERR3335783
ERR4797793	ERR4797909	ERR4798027	ERR4798154	ERR4798273	SRR5341348	ERR4796255
ERR4797794	ERR4797915	ERR4798028	ERR4798155	ERR4798274	SRR5341349	ERR4796268
ERR4797795	ERR4797916	ERR4798029	ERR4798161	ERR4798275	SRR5341350	ERR4796279
ERR4797796	ERR4797917	ERR4798030	ERR4798162	ERR4798277	SRR5341353	ERR4796280
ERR4797798	ERR4797920	ERR4798031	ERR4798166	ERR4798278	SRR5341355	ERR4796283
ERR4797800	ERR4797921	ERR4798032	ERR4798170	ERR4798279	SRR5341357	ERR4796284
ERR4797802	ERR4797922	ERR4798033	ERR4798173	ERR4798281	SRR5341358	ERR4796288
ERR4797803	ERR4797925	ERR4798036	ERR4798174	ERR4798282	SRR5341359	ERR4796290
ERR4797805	ERR4797926	ERR4798037	ERR4798175	ERR4798283	SRR5341361	ERR4796292
ERR4797806	ERR4797927	ERR4798043	ERR4798177	ERR4798286	SRR5341362	ERR4796293
ERR4797807	ERR4797928	ERR4798044	ERR4798178	ERR4798287	SRR5341363	ERR4796295
ERR4797808	ERR4797929	ERR4798045	ERR4798179	ERR4798288	SRR5341364	ERR4796296
ERR4797809	ERR4797930	ERR4798046	ERR4798181	ERR4798290	SRR5341367	ERR4796299
ERR4797811	ERR4797932	ERR4798048	ERR4798183	ERR4798291	SRR5341371	ERR4796301
ERR4797812	ERR4797934	ERR4798052	ERR4798184	ERR4798292	SRR5341372	ERR4796302
ERR4797813	ERR4797935	ERR4798054	ERR4798185	ERR4798295	SRR5341374	ERR4796305
ERR4797814	ERR4797936	ERR4798055	ERR4798186	ERR4798296	SRR5341379	ERR4796308
ERR4797815	ERR4797937	ERR4798056	ERR4798187	ERR4798297	SRR5341382	ERR4796310
ERR4797816	ERR4797938	ERR4798058	ERR4798189	ERR4798301	SRR5341385	ERR4796312
ERR4797818	ERR4797939	ERR4798061	ERR4798190	ERR4798365	SRR5341388	ERR4796313
ERR4797820	ERR4797940	ERR4798063	ERR4798191	ERR4798366	SRR5341389	ERR4796317
ERR4797821	ERR4797941	ERR4798064	ERR4798194	ERR4798370	SRR5341391	ERR4796318
ERR4797822	ERR4797942	ERR4798065	ERR4798195	ERR4798379	SRR5341393	ERR4796323

ERR4796325	ERR4796546	ERR4796747	ERR4797005	ERR4797197	ERR4797361	ERR4797513
ERR4796330	ERR4796551	ERR4796748	ERR4797006	ERR4797198	ERR4797362	ERR4797515
ERR4796332	ERR4796552	ERR4796752	ERR4797009	ERR4797202	ERR4797364	ERR4797517
ERR4796334	ERR4796554	ERR4796753	ERR4797010	ERR4797205	ERR4797365	ERR4797519
ERR4796336	ERR4796556	ERR4796755	ERR4797011	ERR4797206	ERR4797368	ERR4797520
ERR4796338	ERR4796563	ERR4796756	ERR4797013	ERR4797207	ERR4797369	ERR4797527
ERR4796340	ERR4796564	ERR4796759	ERR4797021	ERR4797211	ERR4797371	ERR4797530
ERR4796345	ERR4796567	ERR4796762	ERR4797022	ERR4797212	ERR4797372	ERR4797532
ERR4796347	ERR4796569	ERR4796769	ERR4797023	ERR4797216	ERR4797374	ERR4797533
ERR4796354	ERR4796574	ERR4796770	ERR4797024	ERR4797220	ERR4797375	ERR4797534
ERR4796356	ERR4796580	ERR4796772	ERR4797030	ERR4797222	ERR4797376	ERR4797536
ERR4796358	ERR4796582	ERR4796778	ERR4797032	ERR4797224	ERR4797379	ERR4797538
ERR4796362	ERR4796587	ERR4796783	ERR4797033	ERR4797225	ERR4797381	ERR4797540
ERR4796363	ERR4796591	ERR4796784	ERR4797036	ERR4797226	ERR4797383	ERR4797541
ERR4796364	ERR4796592	ERR4796796	ERR4797038	ERR4797229	ERR4797385	ERR4797543
ERR4796366	ERR4796593	ERR4796799	ERR4797049	ERR4797231	ERR4797386	ERR4797546
ERR4796367	ERR4796598	ERR4796800	ERR4797055	ERR4797236	ERR4797388	ERR4797548
ERR4796373	ERR4796603	ERR4796801	ERR4797057	ERR4797237	ERR4797396	ERR4797554
ERR4796376	ERR4796604	ERR4796806	ERR4797058	ERR4797238	ERR4797397	ERR4797559
ERR4796381	ERR4796606	ERR4796809	ERR4797062	ERR4797239	ERR4797398	ERR4797568
ERR4796386	ERR4796610	ERR4796810	ERR4797063	ERR4797240	ERR4797403	ERR4797569
ERR4796393	ERR4796611	ERR4796811	ERR4797064	ERR4797242	ERR4797404	ERR4797571
ERR4796400	ERR4796614	ERR4796814	ERR4797065	ERR4797243	ERR4797410	ERR4797572
ERR4796401	ERR4796617	ERR4796815	ERR4797067	ERR4797244	ERR4797411	ERR4797574
ERR4796403	ERR4796619	ERR4796819	ERR4797071	ERR4797245	ERR4797416	ERR4797575
ERR4796406	ERR4796620	ERR4796829	ERR4797073	ERR4797249	ERR4797417	ERR4797576
ERR4796407	ERR4796625	ERR4796830	ERR4797076	ERR4797250	ERR4797418	ERR4797583
ERR4796411	ERR4796626	ERR4796834	ERR4797077	ERR4797251	ERR4797420	ERR4797584
ERR4796417	ERR4796630	ERR4796835	ERR4797078	ERR4797253	ERR4797422	ERR4797589
ERR4796419	ERR4796632	ERR4796841	ERR4797079	ERR4797254	ERR4797424	ERR4797590
ERR4796422	ERR4796637	ERR4796842	ERR4797085	ERR4797255	ERR4797425	ERR4797593
ERR4796426	ERR4796638	ERR4796844	ERR4797087	ERR4797256	ERR4797427	ERR4797595
ERR4796428	ERR4796639	ERR4796846	ERR4797089	ERR4797262	ERR4797430	ERR4797598
ERR4796429	ERR4796640	ERR4796848	ERR4797091	ERR4797267	ERR4797431	ERR4797599
ERR4796431	ERR4796642	ERR4796852	ERR4797092	ERR4797270	ERR4797433	ERR4797602
ERR4796433	ERR4796643	ERR4796854	ERR4797098	ERR4797271	ERR4797435	ERR4797603
ERR4796445	ERR4796644	ERR4796856	ERR4797101	ERR4797273	ERR4797437	ERR4797605
ERR4796446	ERR4796650	ERR4796860	ERR4797103	ERR4797276	ERR4797442	ERR4797609
ERR4796453	ERR4796651	ERR4796865	ERR4797104	ERR4797280	ERR4797443	ERR4797610
ERR4796455	ERR4796653	ERR4796866	ERR4797109	ERR4797281	ERR4797444	ERR4797614
ERR4796457	ERR4796656	ERR4796867	ERR4797112	ERR4797282	ERR4797446	ERR4797615
ERR4796459	ERR4796664	ERR4796870	ERR4797116	ERR4797283	ERR4797447	ERR4797616
ERR4796461	ERR4796666	ERR4796873	ERR4797118	ERR4797284	ERR4797448	ERR4797619
ERR4796464	ERR4796668	ERR4796874	ERR4797120	ERR4797286	ERR4797449	ERR4797623
ERR4796465	ERR4796669	ERR4796883	ERR4797123	ERR4797293	ERR4797450	ERR4797624
ERR4796470	ERR4796670	ERR4796886	ERR4797125	ERR4797294	ERR4797452	ERR4797625
ERR4796474	ERR4796671	ERR4796887	ERR4797129	ERR4797298	ERR4797455	ERR4797627
ERR4796475	ERR4796674	ERR4796889	ERR4797134	ERR4797299	ERR4797456	ERR4797628
ERR4796476	ERR4796675	ERR4796899	ERR4797135	ERR4797300	ERR4797460	ERR4797630
ERR4796477	ERR4796678	ERR4796900	ERR4797136	ERR4797306	ERR4797461	ERR4797639
ERR4796480	ERR4796680	ERR4796901	ERR4797138	ERR4797308	ERR4797462	ERR4797646
ERR4796482	ERR4796684	ERR4796902	ERR4797139	ERR4797309	ERR4797463	ERR4797647
ERR4796485	ERR4796685	ERR4796904	ERR4797140	ERR4797312	ERR4797464	ERR4797649
ERR4796489	ERR4796687	ERR4796909	ERR4797142	ERR4797316	ERR4797465	ERR4797650
ERR4796492	ERR4796688	ERR4796911	ERR4797146	ERR4797318	ERR4797470	ERR4797654
ERR4796493	ERR4796691	ERR4796915	ERR4797149	ERR4797320	ERR4797472	ERR4797655
ERR4796494	ERR4796693	ERR4796919	ERR4797153	ERR4797327	ERR4797473	ERR4797656
ERR4796495	ERR4796696	ERR4796920	ERR4797154	ERR4797328	ERR4797475	ERR4797659
ERR4796498	ERR4796705	ERR4796932	ERR4797155	ERR4797330	ERR4797476	ERR4797664
ERR4796500	ERR4796708	ERR4796934	ERR4797159	ERR4797332	ERR4797477	ERR4797667
ERR4796501	ERR4796713	ERR4796935	ERR4797161	ERR4797335	ERR4797480	ERR4797670
ERR4796504	ERR4796716	ERR4796943	ERR4797164	ERR4797336	ERR4797482	ERR4797671
ERR4796507	ERR4796717	ERR4796957	ERR4797165	ERR4797339	ERR4797484	ERR4797675
ERR4796508	ERR4796719	ERR4796970	ERR4797169	ERR4797342	ERR4797487	ERR4797681
ERR4796515	ERR4796720	ERR4796971	ERR4797170	ERR4797344	ERR4797488	ERR4797684
ERR4796520	ERR4796721	ERR4796980	ERR4797176	ERR4797345	ERR4797494	ERR4797685
ERR4796530	ERR4796728	ERR4796981	ERR4797177	ERR4797347	ERR4797495	ERR4797694
ERR4796531	ERR4796731	ERR4796991	ERR4797179	ERR4797348	ERR4797496	ERR4797698
ERR4796535	ERR4796736	ERR4796993	ERR4797188	ERR4797352	ERR4797500	ERR4797699
ERR4796537	ERR4796739	ERR4796994	ERR4797189	ERR4797354	ERR4797505	ERR4797700
ERR4796540	ERR4796741	ERR4797004	ERR4797190	ERR4797357	ERR4797509	ERR4797701

ERR4797706	ERR4797901	ERR4798089	ERR4798294
ERR4797707	ERR4797905	ERR4798092	ERR4798298
ERR4797708	ERR4797907	ERR4798093	ERR4798299
ERR4797718	ERR4797910	ERR4798096	ERR4798300
ERR4797721	ERR4797911	ERR4798097	ERR4798429
ERR4797723	ERR4797912	ERR4798098	ERR4798445
ERR4797724	ERR4797913	ERR4798101	ERR4798449
ERR4797728	ERR4797914	ERR4798103	ERR4798836
ERR4797729	ERR4797918	ERR4798105	ERR4798837
ERR4797730	ERR4797919	ERR4798108	SRR5341282
ERR4797731	ERR4797923	ERR4798109	SRR5341287
ERR4797733	ERR4797924	ERR4798117	SRR5341291
ERR4797734	ERR4797931	ERR4798118	SRR5341297
ERR4797737	ERR4797943	ERR4798123	SRR5341301
ERR4797741	ERR4797947	ERR4798125	SRR5341324
ERR4797743	ERR4797948	ERR4798132	SRR5341331
ERR4797744	ERR4797949	ERR4798137	SRR5341335
ERR4797745	ERR4797950	ERR4798147	SRR5341336
ERR4797746	ERR4797954	ERR4798148	SRR5341337
ERR4797749	ERR4797956	ERR4798156	SRR5341344
ERR4797752	ERR4797958	ERR4798157	SRR5341351
ERR4797761	ERR4797959	ERR4798158	SRR5341352
ERR4797766	ERR4797960	ERR4798159	SRR5341369
ERR4797767	ERR4797964	ERR4798160	SRR5341370
ERR4797768	ERR4797966	ERR4798163	SRR5341376
ERR4797769	ERR4797969	ERR4798164	SRR5341380
ERR4797771	ERR4797976	ERR4798165	SRR5341383
ERR4797773	ERR4797977	ERR4798167	SRR5341386
ERR4797774	ERR4797985	ERR4798168	SRR5341394
ERR4797775	ERR4797989	ERR4798169	SRR5341401
ERR4797781	ERR4797990	ERR4798171	SRR5341424
ERR4797784	ERR4797991	ERR4798172	SRR5341426
ERR4797785	ERR4797996	ERR4798176	SRR5341427
ERR4797792	ERR4798000	ERR4798180	SRR5341431
ERR4797797	ERR4798001	ERR4798182	SRR5341435
ERR4797799	ERR4798002	ERR4798188	SRR5341440
ERR4797801	ERR4798011	ERR4798192	SRR5341443
ERR4797804	ERR4798013	ERR4798193	SRR5341447
ERR4797810	ERR4798014	ERR4798197	SRR5341452
ERR4797817	ERR4798019	ERR4798199	
ERR4797819	ERR4798020	ERR4798203	
ERR4797825	ERR4798021	ERR4798206	
ERR4797832	ERR4798024	ERR4798211	
ERR4797833	ERR4798026	ERR4798213	
ERR4797835	ERR4798034	ERR4798214	
ERR4797837	ERR4798035	ERR4798215	
ERR4797838	ERR4798038	ERR4798217	
ERR4797839	ERR4798039	ERR4798218	
ERR4797845	ERR4798040	ERR4798220	
ERR4797848	ERR4798041	ERR4798222	
ERR4797851	ERR4798042	ERR4798223	
ERR4797855	ERR4798047	ERR4798227	
ERR4797862	ERR4798049	ERR4798233	
ERR4797863	ERR4798050	ERR4798236	
ERR4797866	ERR4798051	ERR4798237	
ERR4797867	ERR4798057	ERR4798241	
ERR4797873	ERR4798059	ERR4798244	
ERR4797877	ERR4798060	ERR4798245	
ERR4797879	ERR4798062	ERR4798253	
ERR4797881	ERR4798066	ERR4798258	
ERR4797882	ERR4798067	ERR4798263	
ERR4797886	ERR4798071	ERR4798266	
ERR4797887	ERR4798072	ERR4798267	
ERR4797888	ERR4798074	ERR4798271	
ERR4797889	ERR4798076	ERR4798272	
ERR4797890	ERR4798077	ERR4798276	
ERR4797891	ERR4798078	ERR4798280	
ERR4797892	ERR4798083	ERR4798284	
ERR4797895	ERR4798084	ERR4798285	
ERR4797899	ERR4798086	ERR4798289	
ERR4797900	ERR4798088	ERR4798293	

Supplementary Table S2: ENA accession numbers and DR type of high-quality WGS datasets (n=1121) from non-endemic region (Netherlands). The annotated assemblies of these WGS datasets were used for Pan-GWAS analysis. ENA accession numbers marked in red are drug-resistant and those in black are sensitive.

ERR3275158	ERR3275573	ERR3275847	ERR3276161	ERR3275198	ERR3275281	ERR3275356
ERR3275183	ERR3275579	ERR3275855	ERR3276172	ERR3275199	ERR3275282	ERR3275357
ERR3275193	ERR3275584	ERR3275860	ERR3276173	ERR3275200	ERR3275283	ERR3275358
ERR3275205	ERR3275589	ERR3275864	ERR3276176	ERR3275202	ERR3275284	ERR3275359
ERR3275207	ERR3275597	ERR3275869	ERR3276190	ERR3275203	ERR3275285	ERR3275360
ERR3275210	ERR3275601	ERR3275870	ERR3276191	ERR3275204	ERR3275286	ERR3275361
ERR3275213	ERR3275604	ERR3275874	ERR3276195	ERR3275206	ERR3275287	ERR3275362
ERR3275222	ERR3275612	ERR3275880	ERR3276202	ERR3275208	ERR3275289	ERR3275363
ERR3275224	ERR3275614	ERR3275881	ERR3276206	ERR3275209	ERR3275290	ERR3275364
ERR3275225	ERR3275616	ERR3275883	ERR3276208	ERR3275211	ERR3275291	ERR3275365
ERR3275231	ERR3275622	ERR3275890	ERR3276229	ERR3275212	ERR3275292	ERR3275366
ERR3275247	ERR3275629	ERR3275891	ERR3276241	ERR3275214	ERR3275293	ERR3275367
ERR3275258	ERR3275639	ERR3275892	ERR3276244	ERR3275216	ERR3275294	ERR3275368
ERR3275263	ERR3275640	ERR3275894	ERR3276259	ERR3275217	ERR3275295	ERR3275370
ERR3275270	ERR3275643	ERR3275914	ERR3276260	ERR3275218	ERR3275296	ERR3275371
ERR3275273	ERR3275649	ERR3275927	ERR3276261	ERR3275219	ERR3275297	ERR3275372
ERR3275274	ERR3275651	ERR3275928	ERR3276262	ERR3275221	ERR3275300	ERR3275373
ERR3275276	ERR3275653	ERR3275930	ERR3276263	ERR3275223	ERR3275301	ERR3275374
ERR3275277	ERR3275661	ERR3275934	ERR3276264	ERR3275226	ERR3275302	ERR3275375
ERR3275288	ERR3275666	ERR3275940	ERR3276265	ERR3275227	ERR3275304	ERR3275377
ERR3275298	ERR3275671	ERR3275944	ERR3276266	ERR3275228	ERR3275305	ERR3275378
ERR3275299	ERR3275672	ERR3275949	ERR3276267	ERR3275229	ERR3275306	ERR3275379
ERR3275303	ERR3275674	ERR3275952	ERR3276268	ERR3275230	ERR3275307	ERR3275380
ERR3275314	ERR3275683	ERR3275954	ERR3276269	ERR3275233	ERR3275308	ERR3275381
ERR3275316	ERR3275689	ERR3275959	ERR3276274	ERR3275234	ERR3275309	ERR3275382
ERR3275322	ERR3275693	ERR3275961	ERR3276277	ERR3275235	ERR3275310	ERR3275383
ERR3275325	ERR3275694	ERR3275963	ERR3276280	ERR3275236	ERR3275311	ERR3275384
ERR3275330	ERR3275695	ERR3275973	ERR3276282	ERR3275237	ERR3275312	ERR3275387
ERR3275336	ERR3275700	ERR3275977	ERR3275156	ERR3275238	ERR3275313	ERR3275389
ERR3275346	ERR3275702	ERR3275979	ERR3275159	ERR3275239	ERR3275315	ERR3275390
ERR3275354	ERR3275703	ERR3275980	ERR3275161	ERR3275240	ERR3275317	ERR3275391
ERR3275369	ERR3275708	ERR3275983	ERR3275162	ERR3275241	ERR3275318	ERR3275392
ERR3275376	ERR3275712	ERR3275987	ERR3275163	ERR3275242	ERR3275319	ERR3275393
ERR3275385	ERR3275716	ERR3275988	ERR3275164	ERR3275243	ERR3275320	ERR3275394
ERR3275386	ERR3275721	ERR3275996	ERR3275165	ERR3275244	ERR3275321	ERR3275395
ERR3275388	ERR3275727	ERR3275997	ERR3275166	ERR3275245	ERR3275323	ERR3275397
ERR3275396	ERR3275730	ERR3276005	ERR3275167	ERR3275246	ERR3275324	ERR3275398
ERR3275418	ERR3275742	ERR3276007	ERR3275168	ERR3275248	ERR3275326	ERR3275399
ERR3275420	ERR3275745	ERR3276011	ERR3275169	ERR3275249	ERR3275327	ERR3275400
ERR3275426	ERR3275750	ERR3276015	ERR3275170	ERR3275250	ERR3275328	ERR3275401
ERR3275432	ERR3275756	ERR3276017	ERR3275171	ERR3275251	ERR3275329	ERR3275402
ERR3275446	ERR3275757	ERR3276019	ERR3275172	ERR3275252	ERR3275331	ERR3275403
ERR3275447	ERR3275758	ERR3276021	ERR3275173	ERR3275253	ERR3275332	ERR3275404
ERR3275448	ERR3275760	ERR3276025	ERR3275174	ERR3275254	ERR3275333	ERR3275405
ERR3275473	ERR3275768	ERR3276026	ERR3275175	ERR3275255	ERR3275334	ERR3275406
ERR3275476	ERR3275774	ERR3276030	ERR3275176	ERR3275256	ERR3275335	ERR3275407
ERR3275481	ERR3275776	ERR3276031	ERR3275177	ERR3275257	ERR3275337	ERR3275408
ERR3275485	ERR3275778	ERR3276042	ERR3275178	ERR3275259	ERR3275338	ERR3275409
ERR3275496	ERR3275780	ERR3276055	ERR3275179	ERR3275260	ERR3275339	ERR3275410
ERR3275507	ERR3275788	ERR3276057	ERR3275180	ERR3275261	ERR3275340	ERR3275411
ERR3275511	ERR3275790	ERR3276064	ERR3275181	ERR3275262	ERR3275341	ERR3275412
ERR3275514	ERR3275792	ERR3276067	ERR3275182	ERR3275264	ERR3275342	ERR3275413
ERR3275518	ERR3275793	ERR3276071	ERR3275184	ERR3275265	ERR3275343	ERR3275414
ERR3275532	ERR3275794	ERR3276072	ERR3275185	ERR3275266	ERR3275344	ERR3275415
ERR3275533	ERR3275796	ERR3276077	ERR3275186	ERR3275267	ERR3275345	ERR3275416
ERR3275534	ERR3275804	ERR3276078	ERR3275187	ERR3275268	ERR3275347	ERR3275417
ERR3275535	ERR3275809	ERR3276081	ERR3275188	ERR3275269	ERR3275348	ERR3275419
ERR3275539	ERR3275810	ERR3276084	ERR3275189	ERR3275271	ERR3275349	ERR3275421
ERR3275556	ERR3275811	ERR3276104	ERR3275191	ERR3275272	ERR3275350	ERR3275422
ERR3275562	ERR3275812	ERR3276107	ERR3275192	ERR3275275	ERR3275351	ERR3275423
ERR3275563	ERR3275831	ERR3276135	ERR3275194	ERR3275278	ERR3275352	ERR3275424
ERR3275568	ERR3275839	ERR3276149	ERR3275195	ERR3275279	ERR3275353	ERR3275425
ERR3275571	ERR3275845	ERR3276158	ERR3275197	ERR3275280	ERR3275355	ERR3275427

ERR3275428	ERR3275512	ERR3275600	ERR3275691	ERR3275791	ERR3275884	ERR3275972
ERR3275429	ERR3275513	ERR3275602	ERR3275692	ERR3275795	ERR3275885	ERR3275974
ERR3275430	ERR3275515	ERR3275603	ERR3275696	ERR3275797	ERR3275886	ERR3275975
ERR3275431	ERR3275516	ERR3275605	ERR3275697	ERR3275798	ERR3275887	ERR3275976
ERR3275433	ERR3275517	ERR3275606	ERR3275698	ERR3275799	ERR3275888	ERR3275978
ERR3275434	ERR3275519	ERR3275607	ERR3275701	ERR3275800	ERR3275889	ERR3275981
ERR3275435	ERR3275520	ERR3275608	ERR3275704	ERR3275801	ERR3275893	ERR3275982
ERR3275436	ERR3275521	ERR3275609	ERR3275705	ERR3275802	ERR3275895	ERR3275984
ERR3275437	ERR3275522	ERR3275610	ERR3275706	ERR3275803	ERR3275896	ERR3275985
ERR3275438	ERR3275523	ERR3275611	ERR3275707	ERR3275805	ERR3275897	ERR3275986
ERR3275439	ERR3275524	ERR3275613	ERR3275710	ERR3275806	ERR3275898	ERR3275989
ERR3275440	ERR3275525	ERR3275615	ERR3275711	ERR3275807	ERR3275899	ERR3275990
ERR3275441	ERR3275526	ERR3275617	ERR3275713	ERR3275808	ERR3275900	ERR3275991
ERR3275442	ERR3275527	ERR3275618	ERR3275714	ERR3275813	ERR3275901	ERR3275992
ERR3275443	ERR3275528	ERR3275619	ERR3275715	ERR3275814	ERR3275902	ERR3275993
ERR3275444	ERR3275529	ERR3275620	ERR3275717	ERR3275815	ERR3275903	ERR3275994
ERR3275445	ERR3275530	ERR3275621	ERR3275718	ERR3275816	ERR3275904	ERR3275995
ERR3275449	ERR3275531	ERR3275623	ERR3275720	ERR3275817	ERR3275905	ERR3275998
ERR3275451	ERR3275536	ERR3275624	ERR3275722	ERR3275818	ERR3275906	ERR3275999
ERR3275452	ERR3275537	ERR3275625	ERR3275723	ERR3275819	ERR3275907	ERR3276000
ERR3275453	ERR3275538	ERR3275626	ERR3275724	ERR3275820	ERR3275908	ERR3276001
ERR3275454	ERR3275540	ERR3275627	ERR3275725	ERR3275821	ERR3275909	ERR3276002
ERR3275455	ERR3275541	ERR3275628	ERR3275726	ERR3275822	ERR3275910	ERR3276003
ERR3275456	ERR3275542	ERR3275630	ERR3275728	ERR3275823	ERR3275911	ERR3276004
ERR3275457	ERR3275543	ERR3275631	ERR3275729	ERR3275824	ERR3275912	ERR3276006
ERR3275458	ERR3275544	ERR3275632	ERR3275731	ERR3275825	ERR3275913	ERR3276008
ERR3275459	ERR3275545	ERR3275633	ERR3275732	ERR3275826	ERR3275915	ERR3276009
ERR3275460	ERR3275546	ERR3275634	ERR3275733	ERR3275827	ERR3275916	ERR3276010
ERR3275461	ERR3275547	ERR3275635	ERR3275734	ERR3275828	ERR3275917	ERR3276013
ERR3275462	ERR3275548	ERR3275636	ERR3275735	ERR3275829	ERR3275918	ERR3276014
ERR3275463	ERR3275549	ERR3275637	ERR3275736	ERR3275830	ERR3275919	ERR3276016
ERR3275464	ERR3275550	ERR3275638	ERR3275737	ERR3275832	ERR3275920	ERR3276018
ERR3275465	ERR3275551	ERR3275641	ERR3275738	ERR3275833	ERR3275921	ERR3276020
ERR3275466	ERR3275552	ERR3275642	ERR3275739	ERR3275834	ERR3275922	ERR3276022
ERR3275467	ERR3275553	ERR3275644	ERR3275740	ERR3275835	ERR3275923	ERR3276023
ERR3275468	ERR3275554	ERR3275645	ERR3275741	ERR3275836	ERR3275924	ERR3276024
ERR3275469	ERR3275555	ERR3275646	ERR3275743	ERR3275837	ERR3275925	ERR3276027
ERR3275470	ERR3275557	ERR3275647	ERR3275744	ERR3275838	ERR3275926	ERR3276028
ERR3275471	ERR3275558	ERR3275648	ERR3275746	ERR3275840	ERR3275929	ERR3276029
ERR3275472	ERR3275559	ERR3275650	ERR3275747	ERR3275841	ERR3275931	ERR3276032
ERR3275474	ERR3275560	ERR3275652	ERR3275748	ERR3275842	ERR3275932	ERR3276033
ERR3275475	ERR3275561	ERR3275654	ERR3275749	ERR3275843	ERR3275933	ERR3276034
ERR3275477	ERR3275564	ERR3275655	ERR3275751	ERR3275844	ERR3275935	ERR3276035
ERR3275479	ERR3275565	ERR3275656	ERR3275752	ERR3275846	ERR3275936	ERR3276036
ERR3275480	ERR3275566	ERR3275657	ERR3275753	ERR3275848	ERR3275937	ERR3276037
ERR3275482	ERR3275567	ERR3275658	ERR3275754	ERR3275849	ERR3275938	ERR3276038
ERR3275483	ERR3275569	ERR3275659	ERR3275755	ERR3275850	ERR3275939	ERR3276039
ERR3275484	ERR3275570	ERR3275660	ERR3275759	ERR3275851	ERR3275941	ERR3276040
ERR3275486	ERR3275572	ERR3275662	ERR3275761	ERR3275852	ERR3275942	ERR3276041
ERR3275487	ERR3275574	ERR3275663	ERR3275762	ERR3275853	ERR3275943	ERR3276043
ERR3275488	ERR3275575	ERR3275664	ERR3275763	ERR3275854	ERR3275945	ERR3276044
ERR3275489	ERR3275576	ERR3275665	ERR3275764	ERR3275856	ERR3275946	ERR3276045
ERR3275490	ERR3275577	ERR3275667	ERR3275765	ERR3275857	ERR3275947	ERR3276046
ERR3275491	ERR3275578	ERR3275668	ERR3275766	ERR3275858	ERR3275948	ERR3276047
ERR3275492	ERR3275580	ERR3275669	ERR3275767	ERR3275859	ERR3275950	ERR3276048
ERR3275493	ERR3275581	ERR3275670	ERR3275769	ERR3275861	ERR3275951	ERR3276049
ERR3275494	ERR3275582	ERR3275673	ERR3275770	ERR3275862	ERR3275953	ERR3276050
ERR3275495	ERR3275583	ERR3275675	ERR3275771	ERR3275863	ERR3275955	ERR3276051
ERR3275497	ERR3275585	ERR3275676	ERR3275772	ERR3275865	ERR3275956	ERR3276052
ERR3275498	ERR3275586	ERR3275677	ERR3275773	ERR3275866	ERR3275957	ERR3276053
ERR3275499	ERR3275587	ERR3275678	ERR3275775	ERR3275867	ERR3275958	ERR3276054
ERR3275500	ERR3275588	ERR3275679	ERR3275777	ERR3275868	ERR3275960	ERR3276056
ERR3275501	ERR3275590	ERR3275680	ERR3275779	ERR3275871	ERR3275962	ERR3276058
ERR3275502	ERR3275591	ERR3275681	ERR3275781	ERR3275872	ERR3275964	ERR3276059
ERR3275503	ERR3275592	ERR3275682	ERR3275782	ERR3275873	ERR3275965	ERR3276060
ERR3275504	ERR3275593	ERR3275684	ERR3275783	ERR3275875	ERR3275966	ERR3276061
ERR3275505	ERR3275594	ERR3275685	ERR3275784	ERR3275876	ERR3275967	ERR3276062
ERR3275506	ERR3275595	ERR3275686	ERR3275785	ERR3275877	ERR3275968	ERR3276063
ERR3275508	ERR3275596	ERR3275687	ERR3275786	ERR3275878	ERR3275969	ERR3276065
ERR3275509	ERR3275598	ERR3275688	ERR3275787	ERR3275879	ERR3275970	ERR3276066
ERR3275510	ERR3275599	ERR3275690	ERR3275789	ERR3275882	ERR3275971	ERR3276068

ERR3276069
ERR3276070
ERR3276073
ERR3276074
ERR3276075
ERR3276076
ERR3276079
ERR3276080
ERR3276082
ERR3276083
ERR3276085
ERR3276086
ERR3276087
ERR3276088
ERR3276089
ERR3276090
ERR3276091
ERR3276092
ERR3276093
ERR3276094
ERR3276095
ERR3276096
ERR3276097
ERR3276098
ERR3276099
ERR3276100
ERR3276101
ERR3276102
ERR3276103
ERR3276105
ERR3276106
ERR3276108
ERR3276109
ERR3276110
ERR3276111
ERR3276112
ERR3276113
ERR3276114
ERR3276115
ERR3276116
ERR3276117
ERR3276118
ERR3276119
ERR3276120
ERR3276121
ERR3276122
ERR3276123
ERR3276124
ERR3276125
ERR3276126
ERR3276127
ERR3276128
ERR3276129
ERR3276130
ERR3276131
ERR3276132
ERR3276133
ERR3276134
ERR3276136
ERR3276137
ERR3276138
ERR3276139
ERR3276140
ERR3276141
ERR3276142
ERR3276143
ERR3276144
ERR3276145
ERR3276146
ERR3276147
ERR3276148

ERR3276150
ERR3276151
ERR3276152
ERR3276153
ERR3276154
ERR3276155
ERR3276156
ERR3276157
ERR3276159
ERR3276160
ERR3276162
ERR3276163
ERR3276164
ERR3276165
ERR3276166
ERR3276167
ERR3276168
ERR3276169
ERR3276170
ERR3276171
ERR3276174
ERR3276175
ERR3276177
ERR3276178
ERR3276179
ERR3276180
ERR3276181
ERR3276182
ERR3276183
ERR3276184
ERR3276185
ERR3276186
ERR3276187
ERR3276188
ERR3276189
ERR3276192
ERR3276193
ERR3276194
ERR3276196
ERR3276197
ERR3276198
ERR3276199
ERR3276200
ERR3276201
ERR3276203
ERR3276204
ERR3276205
ERR3276207
ERR3276209
ERR3276210
ERR3276211
ERR3276212
ERR3276213
ERR3276214
ERR3276215
ERR3276216
ERR3276217
ERR3276218
ERR3276219
ERR3276220
ERR3276221
ERR3276222
ERR3276223
ERR3276224
ERR3276225
ERR3276226
ERR3276227
ERR3276228
ERR3276230
ERR3276231
ERR3276232

ERR3276233
ERR3276234
ERR3276235
ERR3276236
ERR3276237
ERR3276238
ERR3276239
ERR3276240
ERR3276242
ERR3276243
ERR3276245
ERR3276246
ERR3276247
ERR3276248
ERR3276249
ERR3276250
ERR3276251
ERR3276252
ERR3276253
ERR3276254
ERR3276255
ERR3276256
ERR3276257
ERR3276258
ERR3276270
ERR3276271
ERR3276272
ERR3276273
ERR3276275
ERR3276276
ERR3276278
ERR3276281
ERR3276283
ERR3276284
ERR3276285
ERR3276286
ERR3276287
ERR3276288
ERR3276289
ERR3276290
ERR3276291

Supplementary Table S3: Genes that showed significant (BH adjusted P < 0.05) selective presence/absence in drug-resistant Mtb isolates from non-endemic region (Netherlands). A positive $Log_{10}OR$ score indicates a positive association and a negative $Log_{10}OR$ score indicates a negative association with drug-resistant Mtb isolates, respectively.

Locus tag	Gene name	$Log_{10}OR$
Rv1510	hypothetical protein	-2.05
Rv1256c	cytochrome P450 Cyp130	-2.05
Rv1508A	GDP-D-mannose dehydratase GmdA	-2.05
Rv2348c	hypothetical protein	-2.05
Rv1515c	transferase;sugar transferase	-2.05
Rv1514c	glycosyltransferase	-2.05
Rv1507c	hypothetical protein	-2.05
Rv1508c	membrane protein	-2.05
Rv1257c	hypothetical protein;oxidoreductase	-2.05
Rv1509	hypothetical protein;Ubiquinone biosynthesis O-methyltransferase	-2.05
Rv1512	nucleotide-sugar epimerase EpiA	-2.05
Rv1513	hypothetical protein;2-O-methyltransferase NoeI	-2.03
Rv1510	integral membrane protein;hypothetical protein	-1.91
Rv1516c	sugar transferase;hypothetical protein	-1.77
Rv1506c	Trans-aconitate 2-methyltransferase;hypothetical protein	-1.75
Rv3900c	hypothetical protein	-1.49
Rv1255c	HTH-type transcriptional regulator	-1.37
Rv3120	methyltransferase	-1.14
Rv3889c	membrane protein	-1.13
Rv3121	cytochrome P450 Cyp141	-1.12
Rv3618	monooxygenase	-1.09
Rv3622c	PE family protein PE32	-1.09
Rv0394c	hypothetical protein	-1.06
Rv2073c	oxidoreductase	-1.05
Rv2074	pyridoxamine 5'-phosphate oxidase	-1.05
Rv0257	hypothetical protein	-1.04
Rv1555	fumarate reductase membrane anchor subunit	-1.04
Rv1971	Mce family lipoprotein LprM	-0.99
Rv1977	hypothetical protein;Protease HtpX	-0.96
Rv1974	membrane protein	-0.89
Rv1967	Mce family protein Mce3B;Mce associated membrane protein	-0.89
Rv1973	Mce associated membrane protein;hypothetical protein	-0.89
Rv1975	hypothetical protein	-0.89
Rv1976c	hypothetical protein	-0.88
Rv2650c	prophage protein;hypothetical protein	-0.54
Rv2656c	prophage protein	-0.53
Rv2651c	prophage protease;phage prohead protease	-0.53
Rv2658c	prophage protein	-0.52
Rv2655c	prophage protein	-0.52
Rv2646	integrase	-0.52
Rv2645	hypothetical protein	-0.52
Rv2653c	toxin	-0.52
Rv2659c	prophage integrase	-0.52
Rv2657c	prophage protein	-0.52
Rv2654c	antitoxin	-0.50
Rv3829c	dehydrogenase	-0.50
Rv0073	glutamine ABC transporter ATP-binding protein;hypothetical protein	-0.50
Rv0071	maturase	-0.47
Rv2652c	prophage protein	-0.43
Rv2816c	CRISPR-associated endoribonuclease Cas2	-0.42
Rv3347c	hypothetical protein	-0.40
Rv2817c	CRISPR-associated endonuclease Cas1	-0.40
Rv3347c	hypothetical protein	-0.39
Rv2292c	hypothetical protein	-0.36
Rv3725	hypothetical protein	-0.36
Rv3433c	hypothetical protein	-0.33
Rv2647	phage protein;hypothetical protein	-0.31
Rv3433c	hypothetical protein	-0.26
Rv3433c	hypothetical protein	-0.26

Locus tag	Gene name	$Log_{10}OR$
Rv3919c	16S rRNA (guanine(527)-N(7))-methyltransferase RsmG	2.11
Rv1257c	HTH-type transcriptional regulator	1.75
Rv3901c	hypothetical protein	1.49
Rv1809	hypothetical protein	1.45
Rv3829c	dehydrogenase;hypothetical protein	1.41
Rv3331	hypothetical protein	1.24
Rv0279c	PE-PGRS family protein PE_PGRS4	0.79
Rv2818c	hypothetical protein	0.75
Rv3084	acetyl-hydrolase LipR	0.51
Rv3083	FAD-containing monooxygenase MymA	0.51
Rv0072	hypothetical protein	0.48
Rv3533c	hypothetical protein	0.36
Rv2946c	hypothetical protein	0.33
Rv2487c	hypothetical protein	0.30
Rv1946c	hypothetical protein	0.25
